# *FUT2* non-secretor status is associated with altered susceptibility to symptomatic enterotoxigenic *Escherichia coli* infection in Bangladeshis

**DOI:** 10.1038/s41598-017-10854-5

**Published:** 2017-09-06

**Authors:** Lynda Mottram, Gudrun Wiklund, Göran Larson, Firdausi Qadri, Ann-Mari Svennerholm

**Affiliations:** 1 Department of Microbiology and Immunology, Institute of Biomedicine, Sahlgrenska Academy at the University of Gothenburg, Gothenburg, Sweden; 2 Department of Clinical Chemistry and Transfusion Medicine, Institute of Biomedicine, Sahlgrenska Academy at the University of Gothenburg, Gothenburg, Sweden; 3 International Centre for Diarrheal Disease Research, Bangladesh (icddr,b), Dhaka, Bangladesh

## Abstract

Polymorphisms of the FUT2 gene alters glycan ABO(H) blood group and Lewis antigen expression (commonly known as non-secretor status) in the small intestinal mucosa. Whilst non-secretor status affects 20% of the population worldwide, it has been reported to be present in up to 40% of all Bangladeshis. Furthermore, Bangladeshi children are reportedly more susceptible to symptomatic enterotoxigenic *Escherichia coli* (ETEC) infection if they are non-secretors. Therefore, in an attempt to identify a non-secretor status genotypic biomarker of altered susceptibility to ETEC infection, we used the 1000 Genomes Project to identify three population related non-synonymous *FUT2* single nucleotide polymorphisms (SNPs). We then assessed the genotypic frequency of these SNPs in Bangladeshi children who had been clinically monitored for ETEC infection. One novel missense *FUT2* SNP, rs200157007-TT and the earlier established rs601338-AA SNP were shown to be causing non-secretor status, with these SNPs being associated with symptomatic but not asymptomatic ETEC infection. Moreover, rs200157007-TT and rs601338-AA were associated with symptomatic but not asymptomatic ETEC infection irrespective of the child’s Lewis secretor status, suggesting *FUT2*, the regulator of Lewis and ABO(H) antigens in the intestinal mucosa, could be a host genotypic feature affecting susceptibility to ETEC infection.

## Introduction

Changes to histo-blood group antigen (HBGA) expression on mucosal glycans could be a major determinant of disease susceptibility, as HBGAs can serve as nutritional sources, receptors and attachment sites for microorganisms, parasites and viruses^1^. In the mucosa, expression of fucosylated ABO(H) and Lewis HBGAs is under the control of the secretor type (*FUT2*) gene. *FUT2* catalyses the transfer of α1,2-fucosyltransferase to Galactose (Gal) residues on the mucin-type glycan chains (e.g. type 1 Galβ1-3GlcNacβ1-R), leading to the expression of H-antigens in the intestinal epithelium as well as in mucosal and salivary secretions. These H-antigens are then further substituted by blood group A and B transferases to form the blood group A and B antigens, as well as the Lewis transferase (α1-3,4 fucosyltransferase, *FUT3*) to produce the Lewis b (Le^b^, Le(a−b+) phenotype) antigens^2,3^.

Whilst people with functional *FUT2* are known as secretors, individuals (approximately 20% of the population worldwide) who inherit null *FUT2* mutations are termed non-secretors. Non-secretors are unable to synthesise and express ABO(H) or Le^b^ antigens on their mucosal glycans due to the *FUT2* mutation, but they can express Lewis a (Le^a^, Le(a+b−) phenotype, α1-3,4 fucosyltransferase) antigens in their mucosa due to the action of *FUT3*^3^. In rare cases, mutations in both *FUT3* alleles can also occur. These individuals are classified as Lewis negative (Le(a−b−) phenotype) irrespective of their *FUT2* secretor status^2^. Both *FUT2* secretor and non-secretor status have been reported to be associated with either protection against or susceptibility to different gastrointestinal infections^1^.

In a previous longitudinal birth cohort (BC) study, we used saliva and blood phenotyping methods to reveal that Lewis Le(a+b−) and Le(a−b−) phenotypes could exists in up to 26% and 15% (respectively) of all Bangladeshi individuals^4,5^. Moreover, Bangladeshi children with the Lewis non-secretor Le(a+b−) phenotype were found to have an increased susceptibility to symptomatic infection caused by enterotoxigenic *Escherichia coli* (ETEC) expressing the colonisation factor antigen I (CFA/I) fimbriae, as well as other related CFA/I ETEC fimbriae such as CS1, CS2, CS4, CS14, CS17, CS19^5^. One plausible explanation for this association is that the CfaB subunit protein of CFA/I has been shown to bind to the Le^a^ antigen^6^.

The *FUT2* polymorphism causing non-secretor status in the Bangladeshi population is currently unknown, even though *FUT2* is known to have significant worldwide ethnic specific polymorphisms. For example, homozygosity of the nonsense *FUT2* mutation rs601338G > A (G428A, W143X, Table 1) causes non-secretor status in 20% of Caucasians and has been found to be associated with Crohn’s disease, inflammatory bowel disease and infections such as *Helicobacter pylori* and certain genotypes of norovirus and rotavirus^7–13^. In East Asia, non-secretor status is caused by a *FUT2* missense mutation known as rs1047781A > T (A385T, IIe129Phe, Table 1) and has been shown to confer susceptibility similar to diseases as the rs601338G > A genotype^14,15^.

**Table 1:** Non-synonymous *FUT2* SNPs identified with the Bangladeshi 1000 Genomes Project dataset.

SNP ID	Position on exon 2	Reference	Alternative	Consequence type^a^	Amino acid change	Genotype: frequency in worldwide population (n = 2,500)			Genotype: frequency in Bangladesh population (n = 86)		
rs601338G > A	49206674	G	A	Stop gained	W154X	GG: 0.50	GA: 0.35	AA: 0.15	GG: 0.57	GA: 0.38	AA: 0.05
rs602662G > A	49206985	G	A	Missense	G258S	GG: 0.50	GA: 0.35	AA: 0.15	GG: 0.57	GA: 0.38	AA: 0.05
rs200157007C > T	49206548	C	T	Missense	P112L	CC: 0.94	CT: 0.05	TT: 0.01	CC: 0.60	CT: 0.35	TT: 0.05
rs1047781A > T	49206631	A	T	Missense	I140F	AA: 0.86	AT: 0.10	TT: 0.04	AA: 0.93	AT: 0.07	TT: 0.00

^a^Mutational consequence predicted using Ensembl variant effect predictor.

In this retrospective study, we investigate if a *FUT2* genetic polymorphism is associated with the phenotypic non-secretor status found in Bangladesh and if this mutation may be related to increased susceptibility to symptomatic ETEC CFA/I infection. Here we present data from the 1000 Genomes Project^16^ demonstrating the prevalence of three non-synonymous *FUT2* SNPs in the Bangladeshi population, with two of these SNPs being associated with symptomatic ETEC infection.

## Results

### Non-synonymous *FUT2* variants were identified in the 86 Bangladeshi individuals sequenced as part of the 1000 Genomes Project

*FUT2* (ENSG00000176920) is located on Chromosome 19 (genetic coordinates; 19:49,199,228–49,209,207 forward strand) of the human genome, and consists of 3 exons with exon 2 encoding for α1,2 fucosyltransferase, the functional precursor of blood group H and Le^b^ antigens that are found ubiquitously on mucosal glycans of the small intestinal tract^7,17^. We have used a human genomic reference dataset known as the 1000 Genomes Project^16^, to identify potential candidate mutational *FUT2* variants that could cause the non-secretor status Le(a+b−) phenotype.

Out of the initial 40 *FUT2* SNPs we identified in the Bangladeshi 1000 Genomes Project dataset (supplementary S2 dataset), three were non-synonymous (Table 1) and were predicted to change the amino acid sequence. One of the SNPs we identified (Table 1), was the stop gained mutation rs601338G-AA that is commonly associated with non-secretor status and altered susceptibility to disease in people of Caucasian decent^7^. However, the missense SNP rs1047781-TT (Table 1) that causes non-secretor status in the South Asian population^14^ was found to be absent in the 86 Bangladeshi individuals that were sequenced as part of the 1000 Genomes Project. The two other non-synonymous SNPs identified in our analysis (Table 1) were rs602662G > A, a SNP often present with rs601338G > A^10^, and a novel missense mutation called rs200157007C > T that appeared to have a higher genotypic frequency in individuals with East Asian decent compared to individuals from other continents (Table 1 and S1 dataset). We therefore examined the allelic and dominant genotypic frequencies of the rs601338G > A, rs602662G > A and rs200157007C > T SNPs in the Bangladeshi population.

### *FUT2* SNPs are associated with non-secretor status and symptomatic ETEC infection in Bangladeshi children

To study if either rs601338G > A, rs602662G > A or rs200157007C > T are associated with non-secretor status and symptomatic ETEC CFA/I infection in Bangladesh, we performed Taqman SNP RT-PCR genotyping analysis using gDNA extracted from human faecal specimens taken from children who had been part of a 24-month prospective community based BC study to determine the incidence of symptomatic ETEC infection in Bangladeshi children^4^. These children had also been phenotyped for their Le(a − b+), Le(a+b−) or Le (a−b−) status using red blood cell agglutination and saliva dot blot Lewis antigen tests^5^.

In total we were successful in extracting non-degraded and non-defragmented human gDNA from as many as 61 of the 143 children (Table 2) which had been previously phenotyped with either Le(a−b+) or Le(a+b−) status^5^. The Lewis phenotypes and the incidence of symptomatic and asymptomatic ETEC infection in these 61 children are shown in Table 2. The Le(a−b−) cohort was excluded from the data analysis as this group contains a mixture of individuals who can be typed as either secretors or non-secretors but are phenotyped as Lewis negative due to a null mutation in *FUT3*^2^.

**Table 2:** Study subjects with their different Lewis blood group phenotypes.

Lewis phenotype^a^	*n*	Symptomatic ETEC infection (*n* = *38*)	Asymptomatic ETEC infection (*n* = *35*)
Le (a−b+)	39 (54%)	47% (18)	60% (21)
Le (a+b−)	22 (30%)	37% (14)	23% (8)

#### Two homozygous non-synonymous *FUT2* SNPs were identified as being associated with the Le(a + b−) phenotype in the Bangladeshi children

We compared the genotypic distribution of the *FUT2* SNPs shown in Table 1 to the Le(a−b+) and Le(a+b−) Lewis phenotypes (Table 2) of the children from the BC study. As shown in Table 3, we found the established nonsense rs601338-AA (*n* = 6: 27%, *P* = 0.0013)^7^ and the novel missense rs200157007-TT (*n* = 8: 36%, *P* = 0.0028) SNP genotypes to be associated with the Le(a+b−) non-secretor phenotype but not with the Le(a−b+) secretor phenotype (*n* = 39 and 37: 0% and 5% respectively) in the children. In contrast, there was no significant allelic or dominant genotypic association of the rs602662G > A genotypes with Lewis secretor or non-secretor status (*P* = 0.3295) in the BC dataset (Table 3).

**Table 3:** The non-synonymous rs601338-AA and rs200157007-TT SNPs are associated with non-secretor status.

SNP name	SNP genotype	Number of children with Lewis phenotype		*p* ^a^
		Non-secretors	Secretors	
		**Le (a+b−) (n = 22)**	**Le(a−b+) (n = 39)**	
rs601338	AA	27% (6)	0% (0)	0.0013
	GG or GA	72% (16)	100% (39)	
		**Le (a+b−) (n = 22)**	**Le(a−b+) (n = 39)**	
rs200157007	TT	36% (8)	5% (2)	0.0028
	CC or CT	64% (14)	95% (37)	
		**Le (a+b−) (n = 15)**	**Le(a−b+) (n = 27)**	
rs602662	AA	20% (3)	7% (2)	0.3295
	GG or GA	80% (12)	93% (25)	

^a^Fisher’s exact test was performed for the relationship between children with the Le(a + b−) and children with the Le(a − b+) phenotypes and the genotypes of the FUT2 SNPS rs601338, rs200157007 and rs602662.

#### **rs601338-AA and rs200157007-TT SNP genotypes are associated with symptomatic ETEC infection and non-secretor status in Bangladeshi children**

Next we compared the distribution of symptomatic and asymptomatic ETEC infection in the BC children with the rs601338G > A and rs200157007C > T genotypes. No significant allelic or dominant genotypic association was observed between either the nonsense rs601338-AA or missense rs200157007-TT SNPs and symptomatic and asymptomatic ETEC infection (P = 0.3067 and P = 0.1985 respectively) (Table 4). However, when children with either of the two SNPs rs601338-AA and rs200157007-TT were combined, they were more prevalent in BC children with symptomatic (*n* = 32; 37%, *P* < 0.05) than asymptomatic (*n* = 29; 14%) ETEC infection.

**Table 4:** rs601338-AA and rs200157007-TT are associated with symptomatic ETEC infection.

Type of ETEC infection	*n*	Number of children with SNP genotype		*p* ^a^
		**rs601338-AA**	**rs601338-GG or GA**	
Symptomatic	32	16% (5)	84% (27)	0.3067
Asymptomatic	29	3% (1)	97% (28)	
		**rs200157007-TT**	**rs200157007-CC or CT**	
Symptomatic	32	22% (7)	78% (25)	0.1985
Asymptomatic	29	10% (3)	90% (26)	
		**rs601338-AA or rs200157007-TT**	**rs601338-GG/GA or rs200157007-CC/CT**	
Symptomatic	32	37% (12)	63% (20)	0.0448
Asymptomatic	29	14% (4)	86% (25)	

^a^Fisher’s exact test was performed to compare the relationship between children with the homozygous non-synonymous SNP genotypes, compared to children with the homozygous wild-type or heterozygous SNP genotypes who had either symptomatic or asymptomatic ETEC infection.

**Figure 1:**
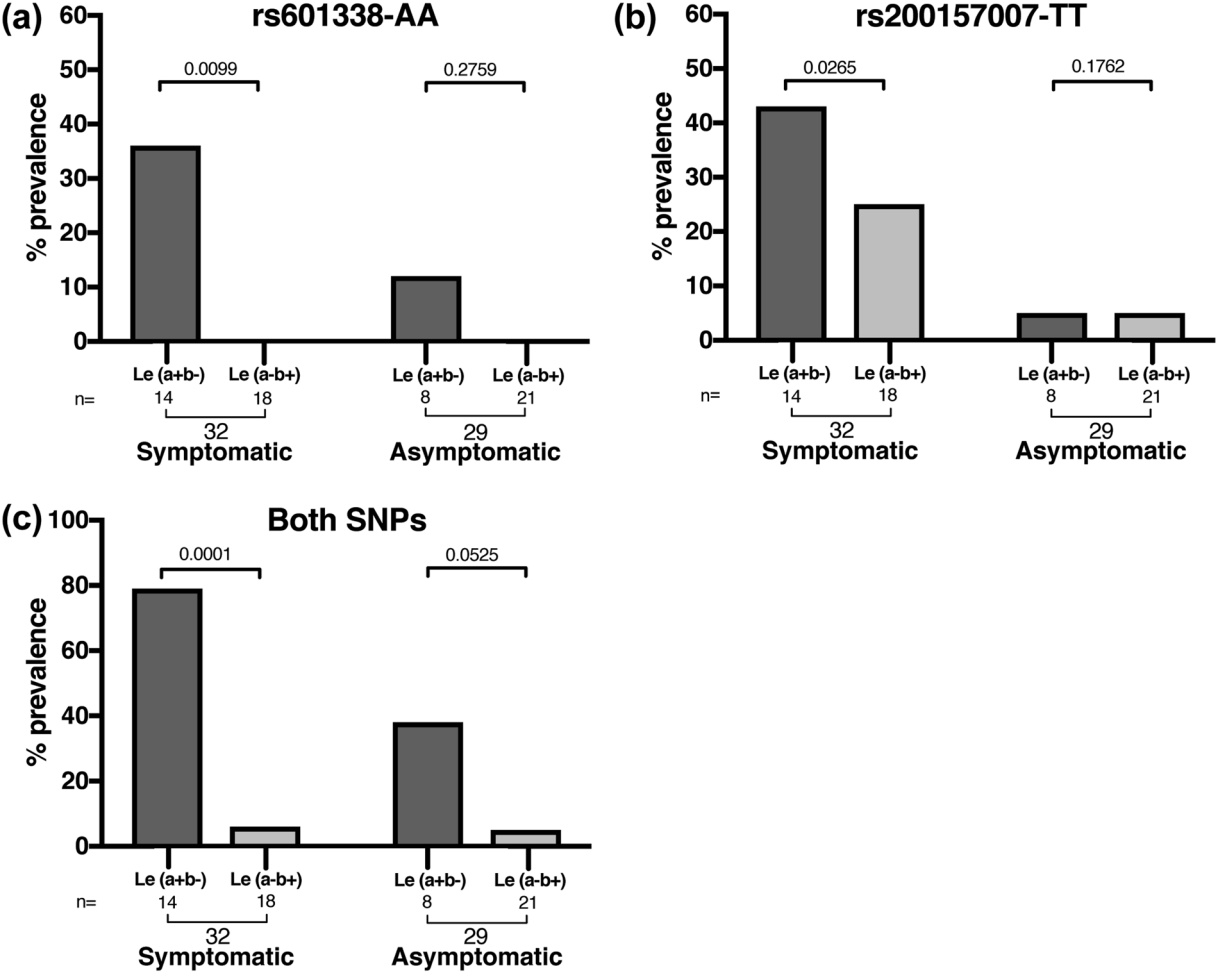
rs601338-AA and rs200157007-TT SNPs are associated with non-secretors status and symptomatic ETEC infection. Association between the Lewis blood group phenotypes Le(a+b−) and Le(a−b+), symptomatic and asymptomatic ETEC infection in the children genotyped with (**a**) the rs601338-AA SNP and (**b**) the rs200157007-TT SNP. (**c**) Shows the combined datasets of the children found with the rs601338-AA and rs200157007-TT SNPs. The Fisher’s exact test was used to compare the relationship between children with the homozygous non-synonymous SNPs genotypes with symptomatic and asymptomatic ETEC infection and their Lewis blood group phenotypes.

Moreover, comparative assessment of the distribution of symptomatic and asymptomatic ETEC infection in the Lewis Le(a−b+) and Le(a+b−) phenotyped BC children revealed a strong association (Fig. 1) between both the rs601338-AA and rs200157007-TT genotypes individually. The rs601338-AA SNP was found to be more prevalent in Le(a+b−) children (*n* = 32; 36%, *P* = 0.0099) than Le(a−b+) children (*n* = 32; 0%) who had symptomatic ETEC infection compared to Le(a + b−) children (*n* = 29; 12%, *P* = 0.2759) and Le(a−b+) children (*n* = 29; 0%) with asymptomatic ETEC infection (Fig. 1). Similarly, the missense rs200157007-TT genotype was more dominant in Le(a+b−) children (*n* = 32; 43%, *P* = 0.0265) than Le(a−b+) children (*n* = 32; 5%) who had symptomatic ETEC infection compared to the Le(a+b−) children (*n* = 29; 25%, *P* = 0.1762) and Le(a−b+) children (*n* = 29; 5%) with asymptomatic ETEC infection.

When studying the relationship between non-secretor status and ETEC CFA/I infection, we observed a trend for higher incidences of infection of ETEC expressing CFA/I as a single fimbriae (26% (*n* = 4) vs 12% (*n* = 1)) or expressing additional fimbriae of the CFA/I group (57% (*n* = 8) vs 37% (*n* = 3)) in BC children with the rs601338-AA or rs200157007-TT SNPs the compared to the children with the rs601338-GG/GA or rs200157007-CC/CT genotypes. However, this relationship was not significant.

## Discussion

To identify a potential non-secretor status (defined by *FUT2*) genetic biomarker in the Bangladeshi population, that is associated with the severity of ETEC infection^2,4,5^, we used the 1000 Genomes Project dataset^16^ to identify three potential *FUT2* SNP candidates that could cause non-secretor status in Bangladeshi individuals. We then performed a retrospective study to determine the prevalence of these SNPs in Bangladeshi children who had been infected with ETEC. These children had previously been part of a two year birth cohort study in Bangladesh and had been diagnosed with either symptomatic or asymptomatic ETEC infection and classified as either secretors (Le(a−b+)) or non-secretors (Le(a+b−)) using blood and saliva phenotyping methods^4,5^.

We strikingly identified strong associations between phenotypic non-secretor status and two of the non-synonymous *FUT2* SNP candidates in the BC children. These were the novel missense *FUT2* SNP rs200157007-TT (*P* = 0.0028) and a previously characterised stop gain *FUT2* SNP often found in individuals of Caucasian decent called rs601338-AA (*P* = 0.0013)^1,7^. Further analysis also revealed rs200157007-TT and rs601338-AA to be associated with symptomatic ETEC infection. We observed rs200157007-TT to be associated with symptomatic (*P* = 0.0028) but not asymptomatic (*P* = 0.1762) ETEC infection in the children who had been phenotyped as non-secretors. Likewise, children with the rs601338-AA SNP and phenotypic non-secretor status, where found to be more likely suffering with symptomatic (*P* = 0.0099) than asymptomatic (*P* = 0.2759) ETEC infection. Overall, rs200157007-TT and rs601338-AA were also found to be associated with symptomatic but not asymptomatic ETEC infection (*P* = 0.0448) irrespective of the child’s phenotypic secretor status, suggesting that *FUT2* could be a host genotypic feature affecting susceptibility to diarrhoeal ETEC disease.

Our previous studies have shown associations between ETEC infection caused by ETEC strains expressing the CFA/I group CF’s and non-secretor status^5^. Using chromatographic techniques we have also demonstrated that the major subunit cfaB of ETEC CFA/I fimbriae can bind to Le^a^ glycophingolipids^6^. However, in this present study and potentially due to the limited number of samples from which we could extract gDNA from, we could only find a trend but not a significant relationship between the rs200157007-TT and rs601338-AA SNPs, non-secretor status and ETEC infection caused by strains expressing CFA/I group CF’s. To address this further we are currently performing additional studies *in-vitro* to characterise the binding of ETEC CFA/I fimbriae to Le^a^ antigens found in the small intestine of humans of non-secretor status.

Polymorphisms such as rs601338 that regulate *FUT2* secretor status, have long been suggested to modulate innate immune responses and may also have a role in human evolutionary survival during different pathogen out breaks^1,18^. As a result, individuals devoid of HBGAs to which a pathogen binds may be protected from the disease. For example, non-secretor status is associated with increased susceptibility to pathogenic *Escherichia coli* urinary tract infections, *Neisseria meningitides*, *Haemopilus influenza* and *Candida albicans* infections^1^. *Helicobacter pylori* on the other hand preferentially infect secretors rather than non-secretors as the bacterium binds to Le^b^ antigens on the gastric epithelial surface^1,11,13^. Interestingly, secretors have also been shown to be more susceptible to certain norovirus and rotavirus genotypes, compared with non-secretors^19^. In particular, secretors are significantly more likely to be infected with P[8] rotavirus genotypes rather than non-secretors. This rotavirus genotype is a major component of licenced rotavirus vaccines and a less prevalent rotavirus genotype in some areas of Bangladesh, but more commonly found in high-income countries^19–21^.

*FUT2* alleles are expressed in small intestinal epithelial cells that are in contact with the external environment and various microorganisms that can bind to these epithelial cells, via glycans that are either precursors of the blood group H antigens or the H antigen itself^1^. It is well known carriers of the nonsense *FUT2* rs601338G-AA SNP do not secrete H-antigen, whilst individuals with the missense *FUT2* rs1047781-TT SNP commonly found in the South Asian population express low levels of H antigen^7,14,19^. The Ganges delta has the lowest prevalence of blood group O individuals worldwide, a host genetic factor associated with an increased risk of severe cholera^22,23^. It is plausible that cholera at least historically has exerted significant selective pressure by causing mortality before reproductive age, thus contributing to the low blood group O individuals in cholera epidemic areas^1,22,24^. Therefore, an association between cholera and HBGA phenotypes could be one possible explanation why there is a high prevalence of non-secretor individuals in Bangladesh at a price of a higher susceptibility to perhaps the more selective ETEC pathogen expressing different colonisation factors^6,24,25^.

In this study we have presented evidence that the *FUT2* gene, which defines secretor status and the expression of the ABO(H) and Lewis HBGAs in small intestinal mucosa may be one of the host genotypic features that determines the outcome of ETEC infection. We show two non-synonymous *FUT2* SNPs; rs200157007-TT and rs601338-AA to be strongly associated with non-secretor status and also symptomatic ETEC infection in Bangladeshi’s. These findings could aid in increasing our understanding of genetic host susceptibility to diarrhoeal disease, and potentially aid our evaluation of host genetic factors to ETEC infection in other ETEC endemic countries worldwide.

## Materials and Methods

### Analysis using 1000 Genomes Project data

The 1000 Genomes Project is a large public catalogue of genetic variants (allele frequency (AF) > 0.01) found in 2,504 human genetic sequences of 26 different populations worldwide. Included in this dataset are 86 genetic sequences from individuals living in Dhaka, Bangladesh (BEB population), who class themselves as Bengali descendants. These study individuals declared themselves as over 18 years of age and healthy at the time of genetic material collection^16^. Interestingly, the 1000 Genomes Project BEB population (86 individuals) has been found to have a distinct genetic grouping, containing a possible genetic admixture of Singapore Indian and Singapore Chinese Indian populations. The most closely genetically related population to the 1000 Genomes Project BEB population was found to be the Gujarati Indians^22^.

All the analysis for this part of the study was performed using the Ensembl genome reference HG19/GRCh37v80. Briefly, the 1000 genomes variant calling file (VCF) of Chromosome 19 (location 49:199,228-49,209,207) and the 1000 genomes sample population mapping file were imputed into the Ensembl AF calculator to predict the total allele count and alternative allele count of all the *FUT2* genetic variants present in each of the 26 populations worldwide^17,26^.

In total 371 worldwide *FUT2* SNPs were identified with an AF > 0.01 (see S1 dataset for further details). The genotype frequency for each variant was calculated manually using the AF values, and the effect of the SNP on gene and protein function was predicted using the Ensembl Variant Effect Predictor (VEP) tool^27^. To confirm analysis findings, the AF of SNPs of interest were further checked on an external human SNP database to the 1000 Genomes Project known as the ExAc browser^28^.

### Patient dataset

From April 2002 to October 2004, a 24-month prospective community based birth cohort (BC) study was conducted in an urban slum in Mirpur, Dhaka, Bangladesh to determine incidence of diarrhoeal disease in children under two years of age^4^. Two years after the completion of the BC study, 179 children who were designated as having ETEC infection were recalled (age, >4 years), and their Lewis phenotypes were determined using Lewis red blood cell agglutination test and also a saliva Lewis dot blot antigen test^5^. Importantly, children who were found to have any other pathogenic infections than ETEC were excluded from our analysis.

### Ethics statement

The study was approved by the Ethics Review Committee of the International Centre for Diarrheal Disease Research, Dhaka, Bangladesh (icddr,b) and informed written consent was obtained from the parents/guardians of all children of the BC study prior to participation. All BC patient data was anonymised for this study. We confirm that all methods were performed according to relevant guidelines and regulations.

### DNA extraction for *FUT2* SNP analysis

Genomic DNA (gDNA) was extracted from frozen faecal specimens collected from the BC children, using the Qiagen QIAamp Fast DNA Stool Mini kit with a few modifications as described by Lindquist *et al*.^29^.

The quantity and quality of each human gDNA extraction was measured using the Qiagen DNA quantimize kit on an AB7500 RT-PCR system. The Qiagen DNA quantimize kit analyses 20 different areas of human genomic loci to determine accurately the amplifiable amount of human DNA in each biological sample. It also provides a quality control (QC) score as indication of DNA sample degradation or defragmentation. Consequently faecal extractions with a low human gDNA content (<1ng) and a low QC quality score (<0.04) were identified and not used for the SNP genotyping analysis.

### SNP Genotyping

Template gDNA extracted from the BC study subjects was genotyped in 96 well plates for the *FUT2* rs200157007C > T, rs601338G > A and rs602662G > A SNPs using their retrospective Taqman chemistry probes (Life technologies, Carlsbad, CA) and the Type-it Fast SNP Probe PCR kit (Qiagen) with the AB7500 RT-PCR system. The total amount of template human gDNA used was 1ng per reaction with the human gDNA quality of each template being >0.04 (as assessed by using the Qiagen DNA quantimize kit, see above for further details).

Each 96 well plate run on the AB7500 RT-PCR system included negative controls containing RNAse/DNase free water only and three different control gDNA templates (quantity = 1ng each) of the rs601338 SNP. These control gDNA templates had been extracted from the blood plasma of healthy donors and had been previously genotyped as either rs601338-GG rs601338-GA or rs601338-AA using PCR, Flow cytometric and hemagglutination methods^30^.

gDNA temples, Taqman probes and the SNP probe PCR master mix was prepared according to the Qiagen manufacturers protocol: SNP Genotyping Using the Type-it Fast SNP probe PCR Master Mix (Drying Out the Template DNA). The RT-PCR cycle programme consisted of a five-minute initial PCR activation step at 95 °C, followed by 40 reaction cycles of 15 seconds at 95 °C and then 30 seconds at 60 °C. After PCR amplification, an endpoint read on the AB7500 RT-PCR instrument was performed to determine the allelic genotype of each sample.

Crucially before the SNP genotyping analysis commenced, NCBI BLAST analysis was performed using the genetic sequences of rs200157007C > T, rs601338G > A and rs602662G > A SNPs. We found no significant similarity between these *FUT2* SNP sequences and the genetic sequences of any pathogens or microbiota that might be found in gDNA extracts from stool samples.

### Statistical Analysis

Statistical analysis was performed using Graph Pad Prism 6. The Fisher’s exact test (2 sided) was used to compare the distribution of each of the *FUT2* SNP genotypes (homozygous wild-type allele genotype and heterozygous allele genotype vs homozygous mutation allele genotype) to the secretor Le(a − b+) or non-secretor Le(a+b−) phenotypes of the symptomatic and asymptomatic ETEC infected children of the BC tudy. Significance was set at a P value of < 0.05.

### Data Availability

All data generated and/or analysed are included in this published article (and its Supplementary Information files).

## Electronic supplementary material

S1 dataset

S2 dataset
