# Author Correction: *FUT2* non-secretor status is associated with altered susceptibility to symptomatic enterotoxigenic *Escherichia coli* infection in Bangladeshis

**DOI:** 10.1038/s41598-018-22786-9

**Published:** 2018-04-11

**Authors:** Lynda Mottram, Gudrun Wiklund, Göran Larson, Firdausi Qadri, Ann-Mari Svennerholm

**Affiliations:** 1 Department of Microbiology and Immunology, Institute of Biomedicine, Sahlgrenska Academy at the University of Gothenburg, Gothenburg, Sweden; 2 Department of Clinical Chemistry and Transfusion Medicine, Institute of Biomedicine, Sahlgrenska Academy at the University of Gothenburg, Gothenburg, Sweden; 3 International Centre for Diarrheal Disease Research, Bangladesh (icddr,b), Dhaka, Bangladesh

Correction to: *Scientific Reports* 10.1038/s41598-017-10854-5, published online 06 September 2017

This Article contains typographical errors in the Acknowledgements section.

“We gratefully thank Ammi Grahm (deceased) of the University of Gothenburg for her generous advice and assistance when this study was being set up.”

should read:

“We gratefully thank Ammi Grahn (deceased) of the University of Gothenburg for her generous advice and assistance when this study was being set up.”

